# Microarray amplification bias: loss of 30% differentially expressed genes due to long probe – poly(A)-tail distances

**DOI:** 10.1186/1471-2164-8-277

**Published:** 2007-08-15

**Authors:** Mirjam C Boelens, Gerard J te Meerman, Johan H Gibcus, Tjasso Blokzijl, H Marike Boezen, Wim Timens, Dirkje S Postma, Harry JM Groen, Anke van den Berg

**Affiliations:** 1 Department of Pathology, University Medical Center Groningen, University of Groningen, Groningen, The Netherlands; 2 Pulmonology, University Medical Center Groningen, University of Groningen, Groningen, The Netherlands; 3 Genetics, University Medical Center Groningen, University of Groningen, Groningen, The Netherlands; 4 Epidemiology, University Medical Center Groningen, University of Groningen, Groningen, The Netherlands

## Abstract

**Background:**

Laser microdissection microscopy has become a rising tool to assess gene expression profiles of pure cell populations. Given the low yield of RNA, a second round of amplification is usually mandatory to yield sufficient amplified-RNA for microarray approaches. Since amplification induces truncation of RNA molecules, we studied the impact of a second round of amplification on identification of differentially expressed genes in relation to the probe – poly(A)-tail distances.

**Results:**

Disagreement was observed between gene expression profiles acquired after a second round of amplification compared to a single round. Thirty percent of the differentially expressed genes identified after one round of amplification were not detected after two rounds. These inconsistent genes have a significant longer probe – poly(A)-tail distance. qRT-PCR on unamplified RNA confirmed differential expression of genes with a probe – poly(A)-tail distance >500 nucleotides appearing only after one round of amplification.

**Conclusion:**

Our data demonstrate a marked loss of 30% of truly differentially expressed genes after a second round of amplification. Therefore, we strongly recommend improvement of amplification procedures and importance of microarray probe design to allow detection of all differentially expressed genes in case of limited amounts of RNA.

## Background

Oligonucleotide microarray technology is a powerful application to measure genome-wide changes in mRNA expression levels in various tissue samples or cell lines. In most studies of tissue samples, whole tissue homogenates were used for RNA isolation. However, use of whole tissue homogenates unavoidably results in averaging of expression levels of different cell types present in the tissue. The expression profile of the cell type of interest will therefore be masked or even lost because of the surrounding cells. To circumvent this problem laser microdissection microscopy (LDM) has become an important tool since it allows isolation of pure cell populations from heterogeneous tissue [1]. The disadvantage of LDM is the low yield of RNA. Currently, a wide range of amplification protocols has been developed to increase the amount of RNA for microarray approaches [2]. The most widely accepted and used protocol for microarray approaches is the T7 RNA-polymerase based linear antisense RNA amplification protocol [3]. Whereas total RNA from whole tissue samples is often amplified one round before hybridization, a second round of amplification is generally performed on total RNA isolated from LDM samples to yield sufficient RNA for microarray approaches.

There is ongoing discussion on the effect of a second round of amplification on the reliability of microarray data. It is known that the amplification procedure can introduce a bias by truncating the length of the amplified transcripts due to inefficient cDNA synthesis. In addition, a second round of cDNA synthesis can result in further truncation of the 5' ends of the transcripts, due to random priming at internal sites for the second strand synthesis [4-8]. However, reproducible results on microarrays were described with application of additional rounds of amplification on low amounts of RNA obtained after microdissection [6,9,10]. In addition, the effects of an additional round of amplification on gene expression profiles have been reported to be minor on the basis of high Pearson correlation coefficients of all genes (0.85 to 0.94) between one and two rounds of amplification [4,10,11] and of consistent numbers of differentially expressed genes detected between one and two rounds of amplification [12].

In this study, we investigated the bias of a second round of amplification on microarray results by comparing two rounds of amplification with one round using the Agilent Whole Human Genome platform. In more detail, we compared the differentially expressed genes identified after both amplification methods. We hypothesized that due to further truncation of RNA during the second round of amplification, a number of differentially expressed genes remains undetected when the corresponding microarray probes are designed too far from the starting point of amplification, the poly(A)-tail. To test this hypothesis, we investigated this probe – poly(A)-tail effect on the identification of genes differentially expressed between two microdissected tissue samples after two rounds of amplification in comparison to genes differentially expressed after one round of amplification. Quantitative RT-PCR (qRT-PCR) was used to validate the differential expression of a selection of these genes on unamplified total RNA from the same microdissected tissue samples.

## Results

### RNA isolation and amplification

**Figure 1:**
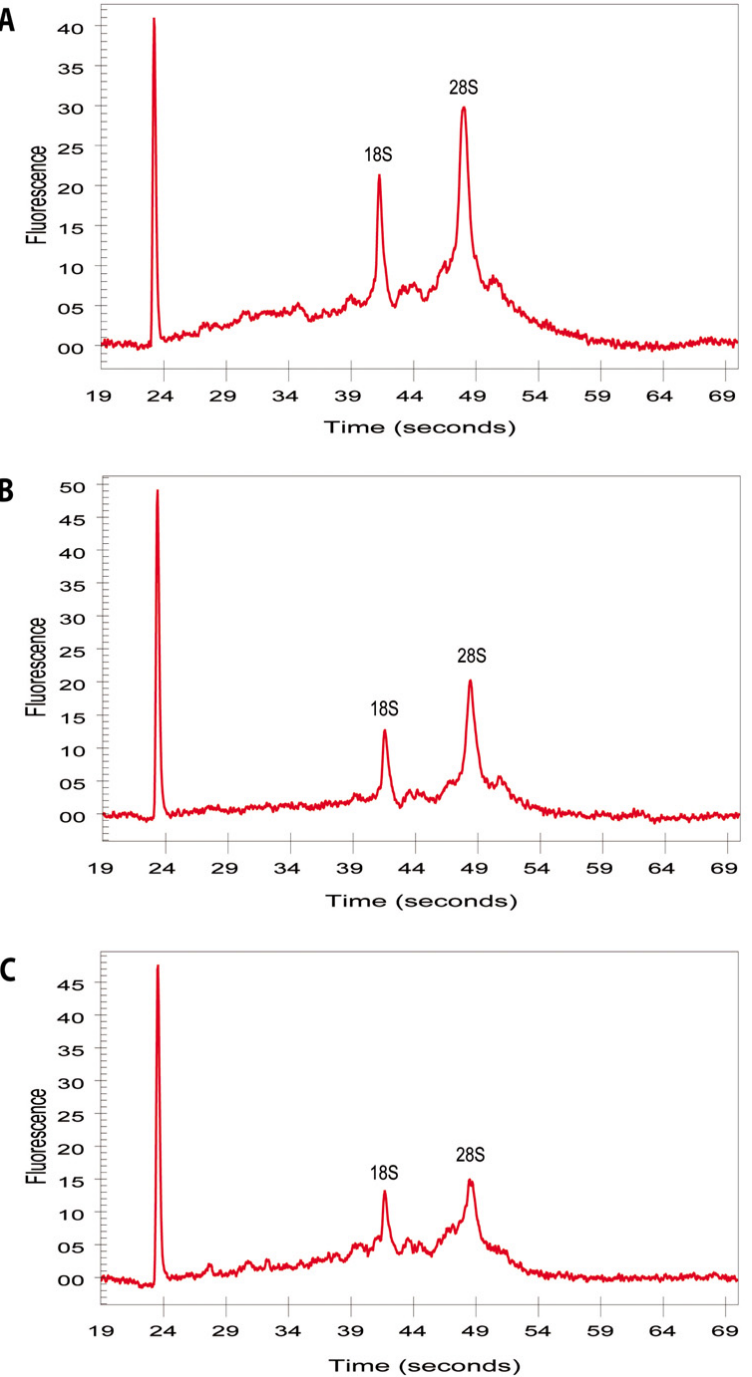
**Electropherograms of microdissected RNA samples**. Beside the marker (left peak) the 18 and 28S peaks are visible. For all samples the 28S is higher than the 18S peak, indicating good quality RNA. **A**, T1, ratio 28S/18S = 1.9; **B**, T2, ratio 28S/18S = 1.9; **C**, B, ratio 28S/18S = 1.8.

**Figure 2:**
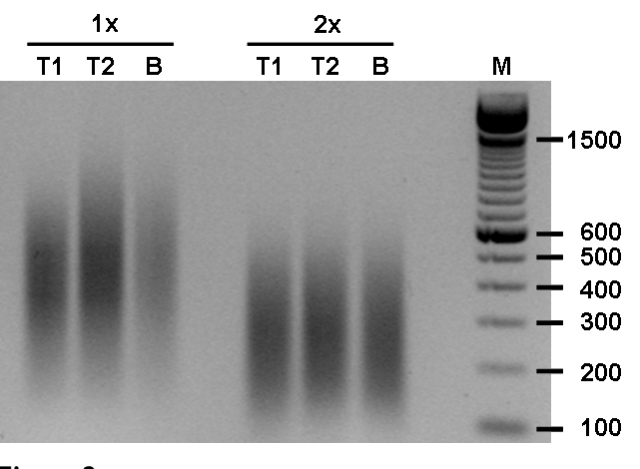
**Agarose gel electrophoresis of amplified RNA samples**. The average sizes of the aRNA of the duplicate tumor samples (T1 and T2) and the bronchus sample (B) is more reduced after two rounds (2x) as compared to the average aRNA sizes after one round (1x) of amplification.

An area of 24.7 × 10^6 ^μm^2 ^of tumor cells was microdissected from the tumor sample and 26.5 × 10^6 ^μm^2 ^of bronchial epithelial cells was microdissected from the bronchus. A quantity of 349 ng of total RNA was isolated from the microdissected tumor cells and 257 ng from the bronchial epithelial cells. RNA quality was sufficient for both samples (Figure 1). The average yield of amplified RNA (aRNA) after a single round of amplification with a 100-ng input of total RNA was 14.3 (13.4–15.2) μg aRNA. From the samples undergoing two rounds of amplification, an input of 25 ng total RNA was used for the 1^st ^round, resulting in an average of 2.7 (2.3–3.0) μg aRNA. According to the manufacturer's instructions 2 μg aRNA was used for the 2^nd ^round of amplification, which yielded an average of 80 (63.8–92.5) μg aRNA. To assess the size of the aRNA after one and two rounds of amplification, we analyzed part of the amplified products on agarose gel (Figure 2). As expected, we observed a marked reduction in size of the aRNA after two rounds of amplification.

### Low agreement between one and two rounds of amplification

**Figure 3:**
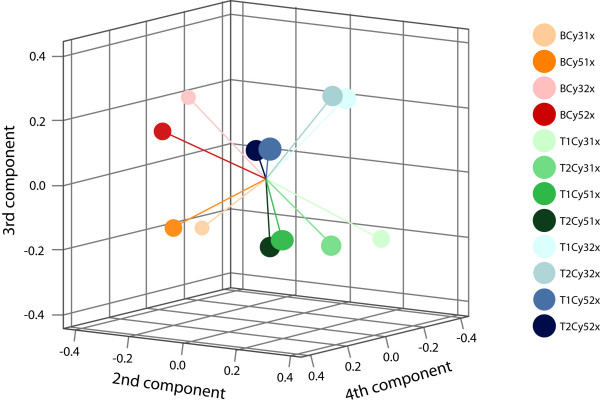
**Principal component analysis of 12 individual hybridizations**. Each hybridization result is illustrated as a colored circle in a 3D graph. The similar the color, the similar the sample. The 2^nd^, 3^rd ^and 4^th ^components are demonstrated on the x-, y- and z-axis respectively with negative and positive values. The 2^nd ^component explains differences in expression of tumor and bronchus (negative values (red-orange) are bronchus, positive values (blue-green) are tumor samples). The 3^rd ^component explains the differences in number of amplification rounds (negative values (orange and green) are one round amplified, positive values (red and blue) are two rounds amplified) and the 4^th ^component explains the labeling type of Cy-dye (negative values (light colored) are Cy3 labeled, positive values (dark colored) are Cy5 labeled samples).

Principal component analysis (PCA) was applied to identify factors that explain the most important variations between our 12 datasets. The first principal component explained 84.8% of the total variance, caused by the commonly observed technical variation between hybridizations. After this variance was subtracted, the largest retained components were visualized in a three-dimensional plot (Figure 3). The 2^nd ^principal component, which explains 5.6% of the total variance, was explained by the difference between tumor and bronchus. The 3^rd ^component (3.5%) was explained by the number of amplification rounds. The 4^th ^component (2.1%) was explained by the difference in Cy-dye label. The subsequent components, less than 1%, could not be explained by a known factor. Unsupervised clustering of the 12 individual datasets showed the same chronological clusters as found with PCA, that is, the largest difference was between tumor and bronchus and the second difference was between the two amplification procedures (data not shown).

**Figure 4:**
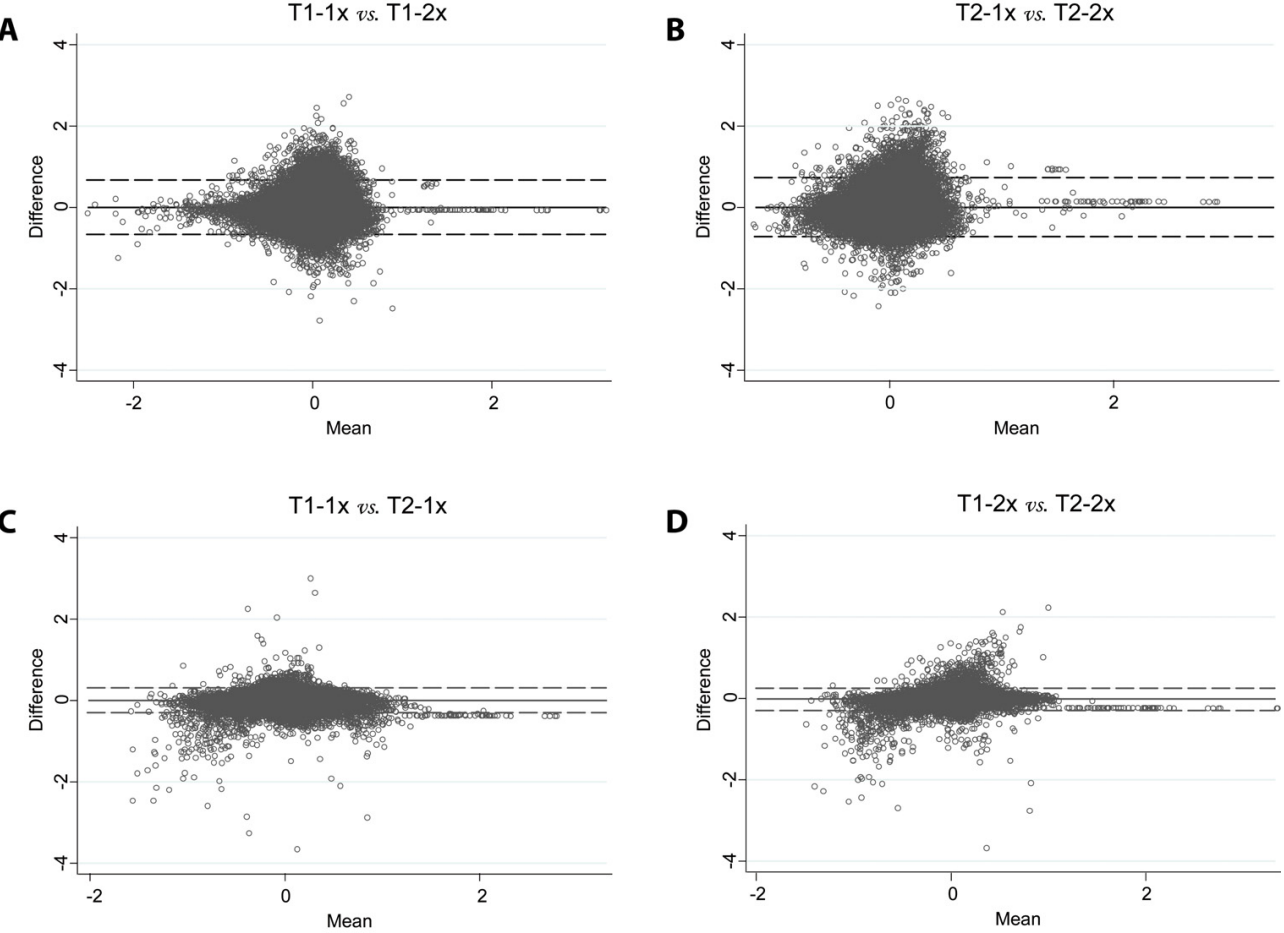
**Bland and Altman plots**. Bland and Altman plots show a large variability between one (1x) and two rounds (2x) of amplification for (**A**) T1 and (**B**) T2 with wide 95% limits of agreement of -0.7 to 0.7. The variability within the same amplification method is much smaller for as well (**C) **1× as (**D**) 2x, both with much narrower 95%-limits of agreement of -0.3 to 0.3. No systematic bias was found. As an example, only Cy5 is shown here. T1-1x, tumor – one round of amplification; T1-2x, tumor – two rounds of amplification; T2-1x, duplicate tumor – one round of amplification; T2-2x, duplicate tumor – two rounds of amplification; dotted line, 95%-limits of agreement.

Bland and Altman plots of the duplicate tumor datasets yielded after one and two rounds of amplification showed a large variability in datasets between these two different amplification methods, with wide 95%-limits of agreement of -0.7 to 0.7 (Figure 4). As a comparison, the control graphs illustrated a much smaller variability within the same amplification method and much narrower 95%-limits of agreement of -0.3 to 0.3. We observed no clear systematic biases between the two methods. The Bradley-Blackwood F test revealed highly significant differences (p < 0.0001) between the datasets of one and two rounds of amplification. The Bradley-Blackwood F test also revealed a highly significant difference (p < 0.0001) between the datasets of the same amplification method. This expected significant difference is in line with the large variability of the first principal component explained by the technical variation commonly observed between microarray hybridizations (84.8% in our study). A high average Pearson correlation coefficient of 0.88 ± 0.01 was calculated between one and two rounds of amplification, which is comparable to previous studies [4,10,11]. The concordance correlation revealed a very low average correlation coefficient between the datasets of one and two rounds of amplification (0.09 ± 0.09) indicating a very low agreement between the two amplification methods. In contrast, the concordance correlation within the same amplification method revealed much higher average coefficients, and thus a much higher agreement (0.63 ± 0.05).

### Inconsistency in differentially expressed genes between one and two rounds of amplification

Differentially expressed genes after one round of amplification were selected at a cut-off level of two times the standard deviation (2×SD) (p < 0.05) according to the formulas described in Methods. 1,312 differentially expressed genes were identified after one round of amplification and 1,349 after two rounds. 931 of these genes (70%) were identified as differentially expressed genes for both amplification approaches. Application of more stringent cut-off levels revealed an average consistency of 368 (75%) and 153 (72%) differentially expressed genes between one and two rounds of amplification at a cut-off level of 3×SD (p < 0.003) and 4×SD (p < 0.0001) respectively. This means that up to 30% of the differentially expressed genes differ between one and two rounds of amplification.

### Loss of differentially expressed genes due to long probe – poly(A)-tail distance

**Figure 5:**
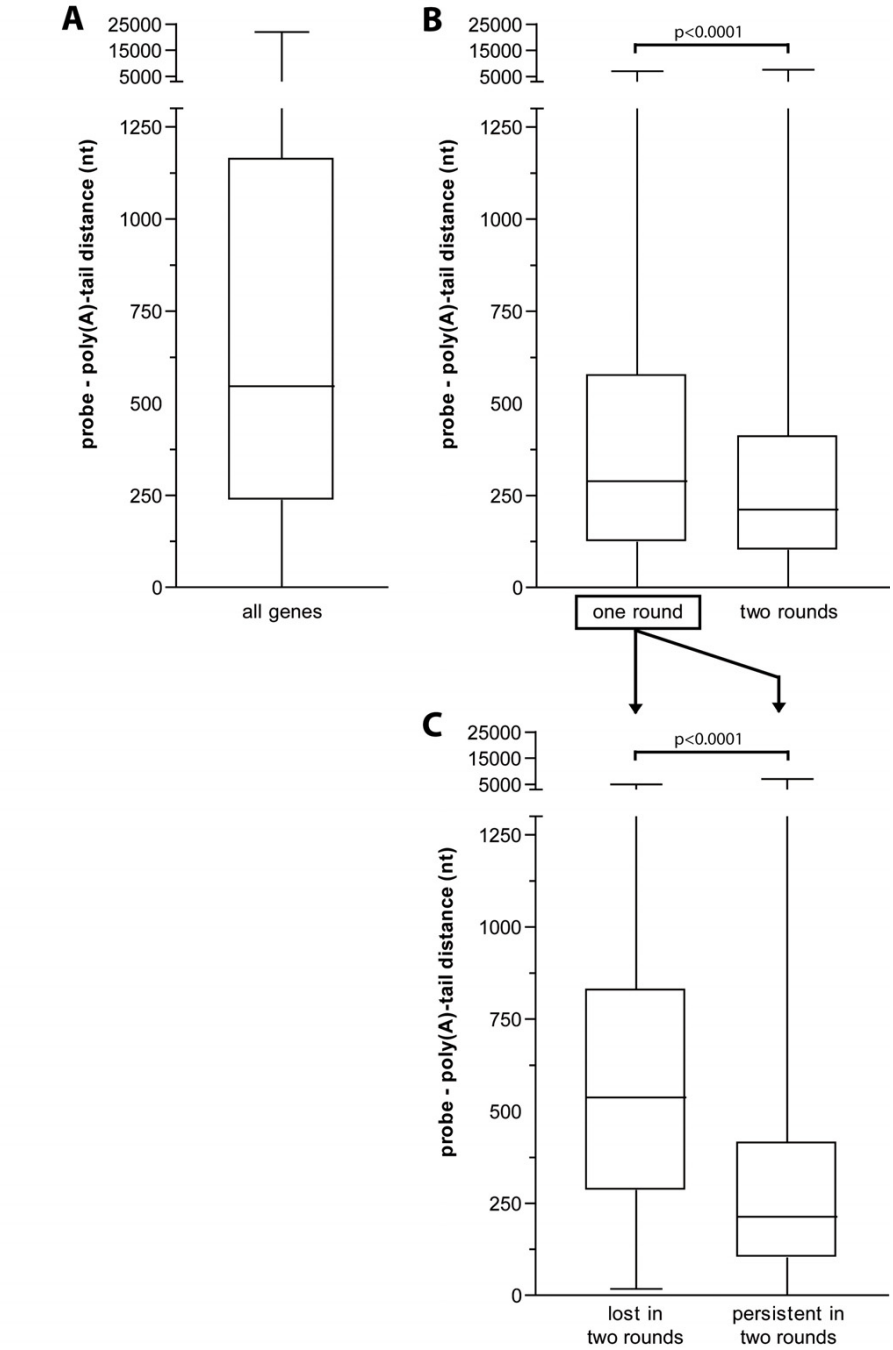
**Probe – poly(A)-tail distance in relation to differentially expressed genes**. **A**, Boxplot of probe – poly(A)-tail distances for 21,103 Agilent WHG probes (median 542 nt, range, 0–22,006) (all genes); **B**, Boxplot of probe – poly(A)-tail distances for the 872 differentially expressed genes after one round (median 289, range, 0–7090) (one round) and 839 differentially expressed genes after two rounds (median 212, range, 0–7621) (two rounds). Mann Whitney analysis revealed a significant decrease of the probe – poly(A)-tail distance after two rounds as compared to one round of amplification (p < 0.0001); **C**, 246 of the 872 genes (~30%) differentially expressed after one round are not identified as being differentially expressed after two rounds of amplification (lost in 2^nd^). These 246 genes have a significant longer probe – poly(A)-tail distance as compared to the remaining 626 genes which were identified as differentially expressed for both one and two rounds of amplification (persistent in 2^nd^) (Mann Whitney: p < 0.0001). Ten of these inconsistent genes, with a probe – poly(A)-tail distance of more than 500 nt, were selected for validation with qRT-PCR;

We investigated if the further truncation of RNA molecules after the second round of amplification can explain the discrepancy observed between genes differentially expressed in one and two rounds of amplification. Therefore, we determined the distance of the 60 mer-oligos (probes) on the Agilent WHG array to the poly(A)-tail of the corresponding genes (see Additional file 1). The 21,103 determined probe – poly(A)-tail distances showed a large variation in distance, with a range from 0 to 22,006 nucleotides (nt) and a median distance of 542 nt (Figure 5A). For the 1,312 genes differentially expressed after one round of amplification, 872 probe – poly(A)-tail distances were determined. These 872 probe – poly(A)-tail distances showed a range from 0 to 7,090 nt with a median of 289 nt, which was significantly different from the probe – poly(A)-tail distances of all genes (p < 0.0001) (Figure 5B). For the 1,349 genes differentially expressed after two rounds of amplification, 839 probe – poly(A)-tail distances were determined. These 839 probe – poly(A)-tail distances showed a range from 0 to 7,621 and a median of 212 nt, which was significantly different from the probe – poly(A)-tail distances of all genes (p < 0.0001) and of differentially expressed genes after one round (p < 0.0001).

246 of the 872 differentially expressed genes identified after one round were not identified as being differentially expressed after two rounds (~30%). These 246 inconsistent genes showed a significant longer probe – poly(A)-tail distance as compared to the remaining 626 consistent differentially expressed genes (Mann Whitney test, p < 0.0001) (Figure 5C).

### Validation of differentially expressed genes by quantitative RT-PCR

Ten of the 246 inconsistent genes (lost in two rounds, Figure 5C) with a probe – poly(A)-tail distance of more than 500 nt were selected for validation with qRT-PCR. qRT-PCR was performed on unamplified microdissected total RNA to verify if these genes were truly differentially expressed between tumor and bronchus (Table 1). As a control, another 10 genes with a probe – poly(A)-tail distance of over 500 nt were selected that were consistent differentially expressed for both one and two rounds (persistent in two rounds, Figure 5C). On average, genes from both amplification groups had comparable probe – poly(A)-tail distances (average, 1,047 bp; range, 573–2,530 bp). The relative expression levels of 18 out of 20 genes showed an up- or down regulation of genes between tumor and bronchus in agreement with the results of the microarray data (Table 1). Two genes, NCOA7 and B2M, showed a similar expression level in tumor and bronchus (0.7 and 1.6 respectively).

**Table 1:** Selected differentially expressed genes and results of validation with qRT-PCR

Gene	Up-/Down- regulation in T versus B	Probe – poly(A)-tail distance (nt)	Relative expression levels (AU)	
			T	B
*lost in two rounds (figure 5C):*				

SLC1A6	Up	803	106	1
PTHLH	Up	1,096	69	1
DKK3	Up	1,104	14	1
CGN	Down	693	1	363
IL18	Down	715	1	7
PTGFR	Down	835	1	40
CCND1	Down	965	1	21
DAF	Down	1,016	1	3
KRT7	Down	1,026	1	35
NCOA7	Down	2,106	1	0.7

*persistent in two rounds (figure 5C):*				

SOST	Up	891	741	1
MCM6	Up	1,039	29	1
SDC2	Up	2,530	108	1
CAPS	Down	573	1	19
B2M	Down	648	1	1.6
NME5	Down	704	1	798
DCN	Down	816	1	19
NS3TP2	Down	820	1	24
GSTA1	Down	824	1	14
UBXD3	Down	1,738	1	72

T, tumor; B, bronchus; nt, nucleotides; AU, arbitrary units.

### Loss of overall signal intensity after two rounds of amplification due to long probe – poly(A)-tail distance

**Figure 6:**
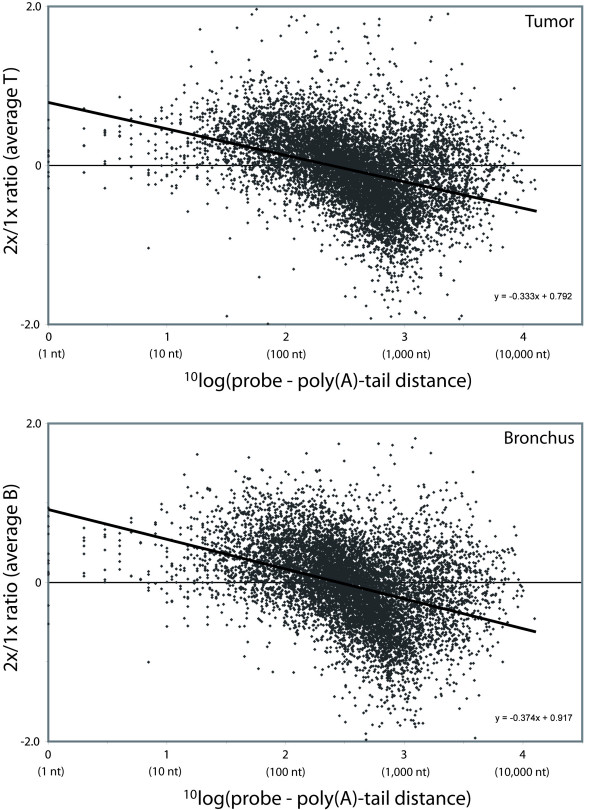
**Probe – poly(A)-tail distance in relation to overall signal intensity**. For each of the 21,103 genes, the average signal intensity was calculated for one and two rounds of amplification for both sample types (T or B) separately. Per sample type, the average signal intensity after one round was subtracted from the average signal intensity after two rounds of amplification (2x/1x ratio). The 2x/1x ratios were plotted against the ^10^log of their corresponding probe – poly(A)-tail distance. A negative regression coefficient for both T and B samples was shown, significantly different from zero (p < 0.0001), that indicates a decrease in overall detection after two rounds of amplification with an increase in probe – poly(A)-tail distance. T, tumor; B, bronchus; 2x, two rounds of amplification; 1x, one round of amplification

We investigated if a long probe – poly(A)-tail distance negatively influences the overall signal intensity of genes after two rounds of amplification. An illustrating graph of this influence is shown in Figure 6. We observed a negative regression coefficient for both T and B samples that were significantly different from zero (p < 0.0001), which indicates that an increase in probe – poly(A)-tail distance results in a decreased signal intensity after two rounds of amplification.

## Discussion

The goal of this study was to evaluate the effect of a second round of T7-RNA polymerase-based amplification, specifically on the identification of differentially expressed genes, using Agilent Whole Human Genome platform. We found that prolonged probe – poly(A)-tail distances are related to a linear decrease in signal intensity. Moreover, we demonstrate a loss of 30% of truly differentially expressed genes after a second round of RNA amplification.

A clear difference between one and two rounds of amplification was found by several statistical tests as discussed below. PCA and unsupervised clustering on the 12 individual hybridization datasets indicated that the number of amplification rounds has an obvious effect on measured signal intensities, which cannot be explained by expression differences (Figure 3). No variance was found that was attributable to the duplicate of the tumor sample, implicating that the same sample, treated independently, had only a minor effect on the outcome of gene expression profiles. This consistency in the duplicates indicates that the amplification procedure itself, as well for one round as for two rounds, resulted in reproducible gene expression profiles similar to previous findings [6,9,10]. Despite the relative high Pearson correlation coefficients between one and two rounds of amplification, comparable to previous studies [4,10,11], concordance correlation revealed very low coefficients, confirming the observations from the Bland and Altman plots. The results of these statistical tests strongly indicate, as expected, a high agreement within the same amplification method but a very low agreement between the two amplification methods.

The microarray technique is usually used by researchers to identify differentially expressed genes between two different RNA samples. Therefore, we investigated the effect of the amplification bias on the identification of differentially expressed genes between the tumor and bronchus LDM samples. Equal numbers of differentially expressed genes were found after one and two rounds of amplification, confirming the previous findings of Wang *et al*. [12]. However, comparison of the list of individual differentially expressed genes demonstrated an inconsistency of up to 30% between one and two rounds of amplification. This percentage remained constant at different significant cut-off levels, indicating that this bias is not due to chance or introduced by noise but is a consistent factor. Comparable observations of a decrease in overlap of differentially expressed genes were shown in previous studies where one or two rounds of amplification were compared with unamplified RNA [13,14].

Although microarray production companies state that their selected probes are located close to the poly(A)-tail of the corresponding transcripts, analysis of the location of probes on the Agilent WHG arrays revealed a wide range in probe – poly(A)-tail distances, from 0 to 22,006 nt with a median of 542 nt. We showed a significant linear decrease in overall signal intensity of genes with an increase in probe – poly(A)-tail distance (Figure 6). This decrease in signal intensity is most likely due to the more prominent truncation of aRNA molecules after two rounds of amplification (Figure 2). Our results are an extension of the findings of McClintick *et al*. who showed a linearly decreased signal after one round of amplification with an increase in probe – poly(A)-tail distances for the probe sets of the Affymetrix arrays [5].

Genes selected as being differentially expressed based on one round of amplification, had significant shorter probe – poly(A)-tail distances as observed for all genes (Figure 5B). In addition, differentially expressed genes after two rounds demonstrated even shorter probe – poly(A)-tail distances (p < 0.0001). Analysis of these 30% inconsistent differentially expressed genes revealed enrichment of genes with long probe – poly(A)-tail distances after one round of amplification, which were lost after two rounds (Figure 5C). The differential expression of these inconsistent genes were validated by qRT-PCR, confirming that a second round of amplification resulted in a 30% reduction of truly differentially expressed genes when probes are located >500 nt from the poly(A)-tail. Thus, it is important to realize that differentially expressed genes can only be identified for those genes that have a short probe – poly(A)-tail distance, especially after two rounds of amplification. Genes with probe-poly(A)-tail distances of more than 500 nt can be falsely classified as being not differentially expressed. Improvement of microarray probe design may help to circumvent the bias introduced by the amplification method, which is currently not optimal for samples with limited amounts of RNA.

We microdissected an area of approximately 25 × 10^6 ^μm^2 ^tumor or bronchial epithelial tissue in approximately 4 hours for each sample. This amount of tissue yielded ~300 ng of total RNA. Although quite labor intensive, this approach allows the isolation of sufficient RNA to apply the more reliable approach including only one round of amplification, since the minimally advised starting amount is 100 ng (Ambion). In general, areas of cell types abundantly present in selected tissue sections, such as squamous cells in non-small cell lung cancer or bronchial epithelial cells in airway tissue, allows isolation of sufficient total RNA in a relatively short time to perform only one round of amplification. However, in the case of single cell research it is clearly not realistic to yield sufficient RNA for one round of amplification.

## Conclusion

This study showed that differentially expressed genes, for which the microarray probe is not selected from a region close to the poly(A)-tail, will remain undetected in gene expression profiles obtained after two rounds of amplification. We demonstrated a loss of 30% of truly differentially expressed genes, which may lead to incomplete data with respect to key pathways in the cell type of interest. This study strongly supports the use of a single round of amplification where possible. Additionally, our results demonstrate the need of improved amplification procedures and innovative microarray probe design to allow reliable detection of differentially expressed genes for samples with limited amounts of RNA.

## Methods

### Tissue specimens

A tumor and a normal bronchus tissue sample were obtained from two patients with squamous cell lung carcinoma (SCC) during surgery at University Medical Center Groningen. The tissues were snap frozen in liquid isopentane, and stored at -80°C until further processing. Written informed consent was obtained from the patients.

### Laser microdissection microscopy

To obtain pure cell populations, we performed laser microdissection microscopy (LDM) [1]. Briefly, frozen sections of 8 μm from lung tissue were mounted on poly-L-lysine coated polyethylene naphthalene membrane-covered slides (P.A.L.M., Bernried, Germany). Air-dried sections were lightly stained with RNase free heamatoxylin (20s), rinsed with DEPC-H_2_O, dehydrated with 100% ethanol and air-dried. Tumor cells were microdissected from tumor sections and histological normal bronchus epithelial cells were microdissected from bronchus sections. An area of approximately 20 to 25 × 10^6 ^μm^2 ^was microdissected by the P.A.L.M. Microlaser Technology system, according to the manufacturer's instructions (P.A.L.M.) and microdissected cells were immediately collected in lysis buffer (Macherey-Nagel, Düren, Germany).

### RNA isolation

Total RNA was isolated and purified from the laser-dissected cells with a Nucleospin RNA II kit (Macherey-Nagel), according to the manufacturer's instructions, including DNase treatment. The quantity of DNA-free total RNA was measured using a Nanodrop ND-1000 spectrophotometer (NanoDrop Technologies, Wilmington, DE). RNA quality was assessed by the presence and ratio of 18S and 28S rRNA bands combined with a low baseline, monitored with RNA 6000 PicoChip (Agilent, Palo Alto, CA) on the 2100 bioanalyzer (Agilent).

### Microarray approach

Agilent Whole Human Genome (WHG) Oligo Microarrays (Agilent, Palo Alto, CA), containing 44,000 60-mer oligonucleotides representing over 41,000 human genes and transcripts, were used to study gene expression levels in laser microdissected squamous lung carcinoma cells and bronchial epithelial cells. A duplicate of the microdissected tumor sample (T1 and T2) and the microdissected bronchial epithelium (B) were amplified for one or two rounds using a total RNA input of 100 ng or 25 ng, respectively. mRNA amplification was performed using MessageAmp II (Ambion, Austin, USA). The main difference between one and two rounds of amplification is the use of different primers. Briefly, the first round of amplification was performed starting with T7 Oligo(dT) primers and the second round of amplification was performed starting with random hexamer primers. mRNA amplification, labeling, hybridization and data extraction were performed at ServiceXS (Leiden, The Netherlands) according to manufacturer's instructions. A dye swap was included and the samples were hybridized using a randomized approach (Table 2), based on the non-competitive conditions of a two-color array [15].

**Table 2:** Randomized hybridization design on Agilent WHG arrays

Data set	Sample	Number of amplification rounds	Dye label	Array number
1	T1	1x	Cy3	3
2	T1	1x	Cy5	1
3	T2	1x	Cy3	1
4	T2	1x	Cy5	2
5	B	1x	Cy3	2
6	B	1x	Cy5	3
7	T1	2x	Cy3	6
8	T1	2x	Cy5	4
9	T2	2x	Cy3	4
10	T2	2x	Cy5	5
11	B	2x	Cy3	5
12	B	2x	Cy5	6

T1, microdissected tumor sample; T2, duplicate microdissected tumor sample; B, microdissected bronchial epithelial sample.

### Statistical analyses of microarray data

To achieve a normal distribution in the data of the Agilent WHG microarray experiments, the median signals of the spot intensities of each hybridization were log2 transformed and standardized to mean = 0 and SD = 1 (log intensities). Principal component analysis (PCA) – a multivariate data analysis tool – was performed to identify factors that explain the largest variance between the individual datasets [16-18]. The first principal component was deducted from the normalized data matrix. This dataset of standardized log intensities were used for further analysis.

We compared the duplicate datasets of the tumor sample of one round of amplification with those of two rounds of amplification. In addition, we included comparisons within the same amplification method as a control. Datasets with the same Cy-dye labeling were compared to preclude possible influence by different Cy-dye labels. First, we constructed Bland and Altman plots to assess agreement between one and two rounds of amplification [19,20]. Any systemic bias or other differences between the two methods can be assessed by eye. In correspondence with the graphical methods of Bland and Altman, we applied the Bradley-Blackwood F test to assess whether the means and differences of expression can be considered to be equal [21]. We determined Pearson correlation coefficients, which measures the degree of linear relationship between two datasets. In addition, we computed concordance correlation coefficients on the selected datasets. The concordance correlation, i.e. rho_concordance with 95% confidence intervals, is a more appropriate way to assess agreement since this test evaluates the degree to which the pairs of observations of the two amplification methods fall on the 45° line through the origin [19,22,23]. STATA was used to construct Bland and Altman plots, perform the Bradley-Blackwood F test and calculate the concordance correlation coefficients. P-values of <0.05 were considered to be statistically significant.

### Selection of differentially expressed genes

To identify differentially expressed genes between the duplicate tumor samples (T1 and T2) and the bronchus sample (B), the ratio was calculated for the two amplification approaches separately from the standardized dataset (see statistical analyses). Briefly, tumor-bronchus ratios (T/B) were determined by subtracting two times the log intensities of the bronchus from the sum of log intensities of the duplicate tumor for both Cy-dyes according to the following formula: (T1+T2) - 2*B. These T/B ratios were calculated for the one and two rounds of amplification samples separately. By comparing the ratios of Cy3 with Cy5 at a threshold of two times the standard deviation (2×SD), consistent differentially expressed genes (p < 0.05) were identified. Specifically, to select upregulated genes in tumor compared with bronchus we used the formula: [T/B(Cy3) > [meanT/B(Cy3) + 2×SD(Cy3)] and T/B(Cy5) > [meanT/B(Cy5) + 2×SD(Cy5)]]. We selected downregulated genes based on the formula: [T/B(Cy3) < [meanT/B(Cy3) - 2×SD(Cy3)] and T/B(Cy5) < [meanT/B(Cy5) - 2×SD(Cy5)]]. Additionally, the number of differentially expressed genes was similarly determined with a threshold at 3×SD (p < 0.003) and 4×SD (p < 0.0001).

### Retrieving probe – poly(A)-tail distances

Since a list of probe – poly(A)-tail distances of the Agilent WHG array is not available, we determined the position of the 60 mer-oligos (probes) in the transcript sequence of their corresponding genes. The sequences of these Agilent WHG probes were retrieved from the website of Agilent [24]. A batch-MegaBLAST was performed within the human refseq mRNA database from NCBI build 36.2 using the probe sequences as query [25]. The retrieved mRNA sequences were filtered to be proven mRNAs (including only "NM_" and "NR_" mRNAs) with a minimal overlap of 57 bases to the input sequence and a minimal identity of 95%. The accession number of the mRNA refseq sequence was used to retrieve the transcript length from the refSeqAli database at the UCSC Table Browser of build Hg18 [26]. 27,590 refseq hits were retrieved for 22,388 Agilent WHG probes. The probe – poly(A)-tail distances of these 22,388 probes were calculated by subtracting the probe position from the transcript length (see Additional file 1). For 21,103 out of these 22,388 probes, a unique probe – poly(A)-tail distance could be determined. The remaining 1,285 probes provided more than one refseq hit with a variance in transcript lengths, which resulted in different probe – poly(A)-tail distances. These 1,285 probes were therefore excluded from further analysis.

### Quantitative RT-PCR

Quantitative RT-PCR (qRT-PCR) was used to validate differential gene expression. Primers of 20 selected genes and the housekeeping gene RNA polymerase II (RPII) were designed using Primer Express software (Applied Biosystems, Foster City, CA). Where possible, primer sequences were chosen to span exon junctions to prevent genomic DNA amplification. Primer sequences of the 20 selected genes and RPII are shown in Table 3. cDNA was synthesized from DNase-treated total RNA isolated from the microdissected tumor and bronchus samples using Superscript II Reverse Transcriptase (Invitrogen, Carlsbad, CA) with random primers (Invitrogen) according to the manufacturer's instructions. qRT-PCR reactions were performed in triplicate in a 20-μl reaction volume containing 1× SYBRgreen mix (Applied Biosystems, Foster City, CA), 900 nmol/l primers and 1 ng cDNA. Reactions were performed on an ABI7900HT Sequence Detection System device (PE Applied Biosystems) using the standard program (10 min at 95°C followed by 40 cycles of 15 s at 95°C, and 60 s at 60°C). Reaction tubes without template cDNA served as negative controls. RPII was used for normalization. The relative amount of transcripts was calculated by subtracting the average Ct value of the reference gene RPII from the average Ct value of the individual selected genes (ΔCt). Next, ΔΔCt values were calculated by subtracting the ΔCt of the tissue type with the lowest expression for that particular gene from the ΔCt of the tissue type with the highest expression (upregulation). Subsequently, relative expression levels were defined as 2^-ΔΔCt^, resulting in the factor of up- or down-regulation between tumor and bronchus.

**Table 3:** Primers of 20 selected differentially expressed genes between tumor and bronchus, used for validation with qRT-PCR

Gene	Accession number	Primers (5'→3')	Product (bp)
SOST	NM_025237	FW-CGCTGCCCATCAGAAAGC REV- CAGGACTAGAAACCACATCTACAGTTG	76
MCM6	NM_005915	FW-GGACCTTTCTTATAGGCTGGTCTTTC REV- GCTCTTTCCCCCCAAACCT	74
SDC2	NM_002998	FW-GAGTGTATCCTATTGATGACGATGACTAC REV- CTCTGGACTCTCTACATCCTCATCAG	77
CAPS	NM_004058	FW-AGGTCACACTGGCGGAATTC REV- GGTCATCATGGCCACGAACT	86
B2M	NM_004048	FW-GAAAAAGTGGAGCATTCAGACTTG REV- ATGATGCTGCTTACATGTCTCGAT	174
NME5	NM_003551	FW-AACTCTGCTTGAAGGACTCACAGA REV- CAGCCAATCAGCTAGCCAAAT	76
DCN	NM_001920	FW-GCTGTCAATGCCATCTTCGA REV- GGGAAGATCCTTTGGCACTTT	71
NS3TP2	NM_023927	FW-TGCTTCCTGAGGCGTTTTG REV- CATGCAATGTCTGGATTCTCATC	76
GSTA1	NM_145740	FW-GCTACTTCCCTGCCTTTGAAAA REV- GCCCGGCTCAGCTTGTT	76
UBXD3	NM_152376	FW-GCCAAGGGACGGACAAGAC REV- AGGCGGAGATGGTATATGATGAG	78
SLC1A6	NM_005071	FW-CTTCAAACAGTTCAAGACGCAGTAC REV- CGGCTCAGACCCGTTCTCT	79
PTHLH	NM_002820	FW-CCGC4CTCAAAAGAGCT715GTGT REV- CGCCGTAAATCTTGGATGGA	72
DKK3	NM_013253	FW-AGGAGCCACGAGTGCATCA REV- GGTGTACTGGAAGCTGGCAAAC	75
CGN	NM_020770	FW-TGAGGAATTCGACAGTGTCTACGA REV- GGTCTGTAGGTTGCTCTCCGTAA	70
IL18	NM_001562	FW-AGGAACCTCAGACCTTCCAGATC REV- CTACTGGTTCAGCAGCCATCTTT	81
PTGFR	NM_000959	FW-GGCCTGGGATGACAAGATGT REV- TTGTGGAGATAAAAGCCAACCA	86
CCND1	NM_053056	FW-CCGTCCATGCGGAAGATC REV- CCTCCTCCTCGCACTTCTGT	69
DAF	NM_000574	FW-TGCCCTAATCCGGGAGAAA REV- GAAGGAGATGGTTGCACCAAA	78
KRT7	NM_005556	FW-GCCACCACCCACAATCACA REV- CTTTCCAGACTGTCTCACTGTCTTG	79
NCOA7	NM_181782	FW-TATGTGGCGGGCACCTGTA REV- CTCTGCTTCCCGGATTCAAG	75
RPII	Housekeeping gene	FW- CGTACGCACCACGTCCAAT REV- CAAGAGAGCCAAGTGTCGGTAA	139

### Calculation of signal intensity ratios between one and two rounds of amplification

To investigate if a long probe – poly(A)-tail distance negatively influences the overall signal intensity of genes after two rounds of amplification, the difference in signal intensity between one and two rounds of amplification (2x/1x ratio) was calculated for each probe. Briefly, genes with no or low expression were first excluded from this analysis by deleting all genes with a median signal intensity smaller than three times the background signal intensity. Next, we calculated the 2x/1x ratio for both samples (T and B) separately by subtracting the average signal intensities of the standardized dataset (see statistical analyses) obtained after one round from those obtained after two rounds of amplification. These 2x/1x ratios were plotted against their corresponding probe – poly(A)-tail distance.

## Authors' contributions

MCB performed the laboratory work (except for qRT-PCR experiments, which were performed by TB), did the data analyses and drafted the manuscript. GJTM performed normalization of the microarray data and principal component analysis. JHG determined the probe – poly(A)-tail distances of the Agilent WHG array. HMB performed the STATA used statistics. WT assessed diagnosis and quality of tissue samples used in this study. WT, DSP, HJMG and AVDB supervised the study, data analyses and editing of the manuscript. All authors read and approved the final manuscript.

## Supplementary Material

Additional file 1 Probe – poly(A)-tail distances of Agilent whole human genome array. For 22,388 probes of the Agilent WHG array, the determined probe – poly(A)-tail distances are listed in this table. Click here for file
